# Bites from the same dog, different outcomes for two patients: a case report

**DOI:** 10.1186/s40249-017-0321-3

**Published:** 2017-07-05

**Authors:** Xue-Yong Huang, Xing-Le Li, Shu-Yu Wu, Yu-Lei Gu, Xin-Jun Lv, John David Klena, Bian-Li Xu

**Affiliations:** 1 Henan Center for Disease Control and Prevention, Zhengzhou, China; 2 Henan Key Laboratory of Pathogenic Microorganisms, Zhengzhou, China; 3 Henan Collaborative Innovation Center of Molecular Diagnosis and Laboratory Medicine, Xinxiang, China; 4 International Emerging Infections Program, United States Centers for Disease Control and Prevention, Beijing, China; 5 Global Disease Detection Branch, Division of Global Health Protection, Center for Global Health, United States Centers for Disease Control and Prevention, Atlanta, GA USA; 6 The First Affiliated Hospital of Zhengzhou University, Zhengzhou, China; 7 Chinese Center for Disease Control and Prevention, Beijing, China

**Keywords:** China, Encephalitis, Neglected diseases, Rabies, Rabies atypical manifestations, Rabies laboratory diagnosis, Rabies postexposure prophylaxis, Zoonosis

## Abstract

**Background:**

Rabies is a serious reemerging zoonosis in China. At present human rabies cases are primarily diagnosed based on clinical presentation.

**Case presentation:**

In August 2012, a woman and her son were attacked by a stray dog in Henan, China. The son received rabies postexposure prophylaxis (wound treatment followed by vaccine, no immunoglobulin), however, the mother did not. Rabies infection was subsequently laboratory confirmed in the mother and she died in December; her son is alive and healthy after 2 years of follow-up.

**Conclusion:**

This report documents that the timely utilization of postexposure prophylaxis is a required measure in preventing rabies after exposure to an animal bite.

**Electronic supplementary material:**

The online version of this article (doi:10.1186/s40249-017-0321-3) contains supplementary material, which is available to authorized users.

## Multilingual abstract

Please see Additional file 1 for translations of the abstract into the five official working languages of United Nations.

## Background

Rabies is a zoonotic infectious disease caused by lyssaviruses. The rabies virus (RV) is the most important lyssavirus and is widely distributed across the globe, with a human mortality rate of almost 100% after the onset of symptoms [1]. China reports the second highest number of human rabies deaths, only after India [2]. Although the number of cases of human rabies has been decreasing since 2007, the disease remains an important public health threat in mainland China [3]. Dogs are the main source of infection and are the primary vector for human rabies in rural China [4].

The purpose of this report is to present the different outcomes in two patients (a mother and a son) bitten by the same dog. The mother did not receive rabies postexposure prophylaxis (PEP) and died of rabies, while her son had no illness or symptoms after receiving the rabies PEP. The report also summarizes how to systematically diagnose a suspected rabies case and a laboratory confirmed rabies case, as well as how to carry out effective prevention and control measures for the disease.

## Case presentation

On 24 November 2012, a 31-year-old woman was diagnosed as a suspected rabies case by the First Affiliated Hospital of Zhengzhou University, which reported the case to the Henan Center for Disease Control and Prevention (CDC). A detailed epidemiological investigation of the suspected rabies case was performed.

The woman was a farmer living in a rural area of Xuchang, Henan Province, China. She lived with her husband and two sons, and said she had not travelled anywhere recently. At about 17:00 on 25 August 2012, the woman and her 7-year-old son were attacked by a stray dog while walking nearby to their village; the woman sustained bites on her right thigh while her son had bites on his left calf. Villagers caught and killed the dog, and it was buried on the village outskirts without laboratory investigation for RV. Believing the wound would result in a severe disability for her child, the mother washed the son’s bites with municipal tap water (without soap) shortly after the incident. On 26 August, she took her son to the healthcare unit in their village, where the boy received a rabies vaccine and completed a full course of standard vaccines (freeze-dried rabies vaccine for human use [Vero cells], Liaoning Chengda Biotechnology Co., Ltd. Dalian City, Liaoning Province, China; a five-dose vaccination regimen on days 0, 3, 7, 14, and 28). In contrast, the mother did not recognize the risk posed to her by the dog bite, and only washed her own wounds the following day, following the doctor’s advice. However, she declined the rabies vaccine for economic reasons. Both the mother’s and son’s wounds were determined by the doctor to constitute category III exposure bites (single or multiple transdermal bites or scratches; contamination of mucous membrane with saliva, i.e. licks), according to the classification criteria of the World Health Organization (WHO) [1]. However, neither the mother nor her son were treated as recommended by the WHO for category III rabies exposure, which requires wound cleaning, rabies vaccination, and direct wound infiltration with rabies immunoglobulin (RIG) [1]. The rabies vaccine is not in the Chinese National Immunization Scheme, so rabies vaccine and RIG are currently provided for a fee in China.

On 20 November 2012, the woman presented with a persistent fever (39 °C), nausea, vomiting, chest tightness, and agitation to the Xiangcheng County People’s Hospital. She received a diagnosis of encephalitis and was treated for 2 days with cefoperazone, sulbactam, levofloxacin, and supportive treatment, including oxygen therapy, intravenous rehydration, and maintenance of adequate electrolyte balance. On 22 November, her symptoms worsened and she developed a mild coma, drooling and melena; she was then transferred to the Xuchang City Central Hospital. On 24 November, she was transferred to the First Affiliated Hospital of Zhengzhou University, where she received a diagnosis of suspected rabies and was treated with symptomatic and supportive therapies. Her condition continued to deteriorate and she died on 6 December 2012.

On 4 December 2012, saliva, serum, and cerebrospinal fluid (CSF) from the patient were collected. On 15 December 2012, a serum sample was collected from her son, who was in good health when sampled. Both sets of specimens were transported under refrigeration to the Henan CDC for testing.

**Fig. 1:**
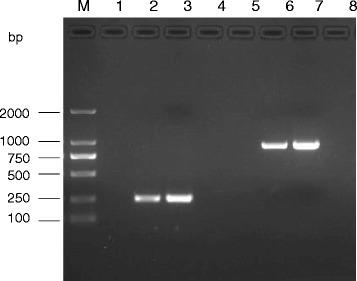
Amplification products of the N and G genes of RV. Lane M, DL2000 DNA Marker; lane 1, N gene amplification product using RNA obtained from patient’s CSF; lane 2, N gene amplification product using RNA obtained from patient’s saliva; lane 3, positive control; lane 4, negative control; lane 5, G gene amplification product using RNA obtained from patient’s CSF; lane 6, G protein gene amplification product using RNA obtained from patient’s saliva; lane 7, positive control; lane 8, negative control

**Fig. 2:**
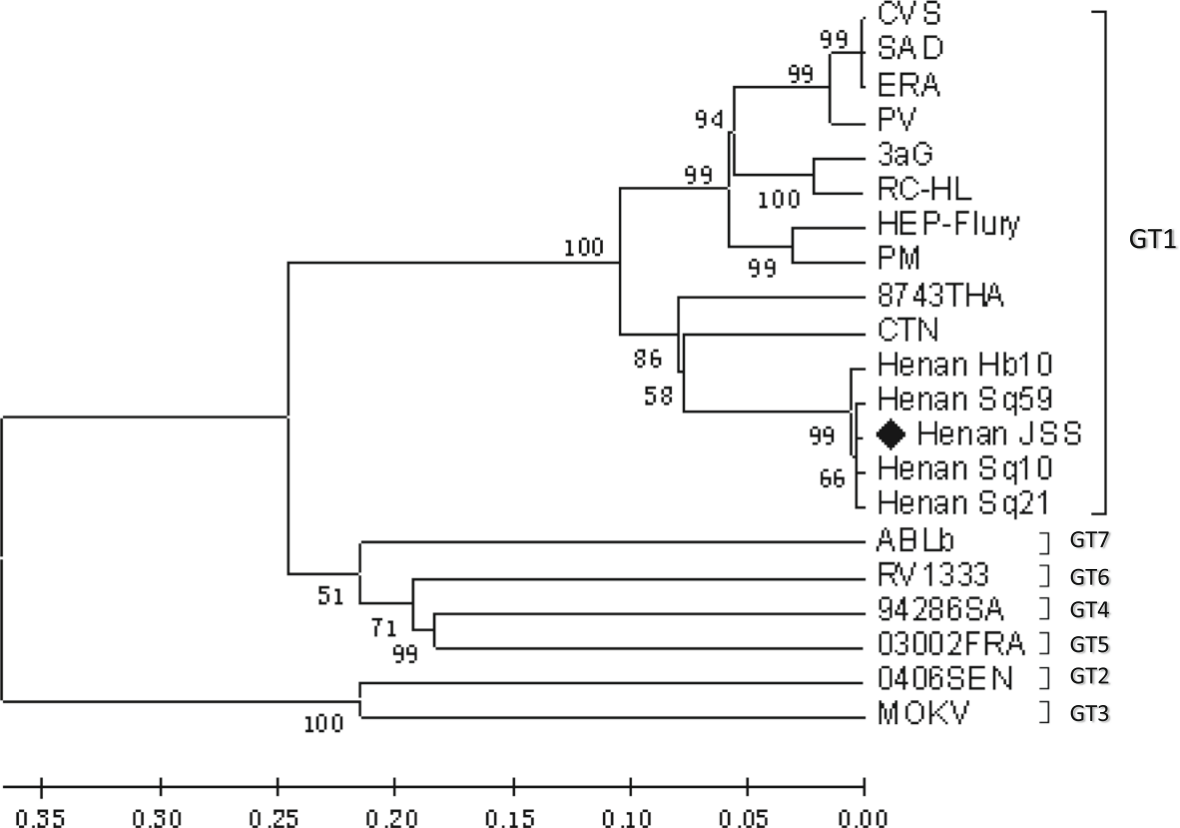
Phylogenetic analysis based on the G gene nucleotide sequences of RV from Henan. With the exception of Henan JSS, the G gene sequences were collected from GenBank. The black spot indicates Henan JSS, the G gene that was amplified by PCR and subsequently sequenced in the present study

Total ribonucleic acid (RNA) was extracted from the CSF and saliva, and reverse transcribed to cDNA. RV N and G genes were amplified using nested polymerase chain reaction (PCR), and negative controls (RNAse-free water) and positive controls (positive CSF specimens were preserved in our laboratory) were included in each set of reactions [5]. Amplification products were detected after electrophoresis using 2% agarose gel. The N and G genes were amplified from the woman patient’s saliva, but not from her CSF (see Figure 1). Amplification products were purified and sequenced using an automated ABI 3730 DNA Sequencer (Applied Biosystems™, Foster City, CA, USA) from Sangon Biotech Co., Ltd. (Shanghai, China). Molecular phylogenetic analysis was conducted using the maximum likelihood method based on the Kimura’s two parameter model with MEGA 5 software (available at: http://mega.software.informer.com/5.0/) [6]. Nucleotide sequence from the female (Henan) patient (Henan JSS, GenBank accession number KP221203) G protein was compared against nucleotide sequences of G protein genes from RV identified in GenBank (Table 1). Henan JSS, along with previous Henan RV strains, the Chinese vaccine strain, and 8743THA (representing strains of RV genotype one) were grouped into GT1 (see Figure 2).

**Table 1:** The GenBank accession numbers of G protein genes from RV obtained in this study

Name of genes	GenBank accession No.	Source	Geographic origin	Year
CVS-11	EU126641	Cattle	France	1882
SAD	M31046	Dog	Alabama, America	1935
ERA	J02293	Dog	Alabama, America	1935
PV	M13215	Cattle	France	1882
3aG	L04522	Dog	Beijing, China	1931
RC-HL	D14873	Dog	France	1882
HEP-Flury	AB085828	Human	Georgia, America	1939
PM	AJ871962	Cattle	France	1882
8743THA	AF401285	Human	Thailand	1983
CTN	AY009100	Human	Shandong, China	1956
Henan Hb10	EU267753	Dog	Henan, China	2008
Henan Sq59	EU267759	Dog	Henan, China	2008
Henan Jss	KP221203	Human	Henan, China	2012
Henan Sq10	EU267756	Dog	Henan, China	2008
Henan Sq21	EU267757	Dog	Henan, China	2008
0406SEN	EU293108	Eidolon helvum	Senegal	1985
MOKV	HM623780	Crocidura sp	Zimbabwe	1981
94286SA	EU293120	Miniopterus	South Africa	1981
03002FRA	EU293109	Eptesicus serotinus	France	2003
RV1333	EF157977	Human	United Kingdom	2002
ABLb	NC_003243	Bat	Australia	1996

The rabies virus neutralizing antibody (RVNA) titers in the sera were assayed by a standard rapid fluorescent focus inhibition test with some modifications [7]. A serum specimen from the patient was collected on day 12 of her illness; her son’s serum was collected 3 months after the bites. Serum RVNA titers of the mother and her son were 0.68 IU/ml and 2.29 IU/ml, respectively. The mother and son were considered positive according to the diagnosis criteria for RVNA reactions (RVNA titers ≥0.05 IU/ml, the WHO recommended protective level) [8].

Because the son did not receive immediate RIG treatment, he remained at possible risk for RV infection [9, 10]. RVNA of the son has been actively monitored; his health condition has been assessed every 6 months post his initial result. Fortunately, the son is alive and healthy after 2 years of follow-up.

## Conclusion

Rabies is a fatal disease, yet it is preventable using proven, effective measures including immediate wound washing with soap and water or other detergents that can kill the virus, vaccine in cases of category III exposures, and wound infiltration with RIG [11–13].

This RV case highlights several important issues in the recognition and treatment of rabies. Prior to transfer to the First Affiliated Hospital of Zhengzhou University, the female patient was hospitalized in a local county and then a municipal hospital, where a clinical diagnosis could not be made with certainty. In these settings, medical personnel have limited experience with rabies recognition and diagnosis.

Secondly, the patient had almost no typical clinical manifestations of rabies, making it more difficult to diagnose her illness. After transfer to the First Affiliated Hospital of Zhengzhou University, the patient was suspected to have a RV infection based on the history of being attacked by a dog and after ruling out other probable causes of craniocerebral injury. Epidemiological history plays an important role in the clinical diagnosis of rabies [14–16]. However, the first two hospitals did not ask about the dog bite history of the patient.

The case reported here highlights the challenge of diagnosing and treating rabies patients in rural areas of China. Public health agencies should increase public awareness around the risk associated with dog bites and improve the application and availability of high-quality anti-rabies vaccines and RIG, in order to prevent rabies infection in China. Controlling rabies through pet vaccination schemes, particularly for dogs, is also an important strategy for reducing the rate of human exposure to rabies.

This report documents the outcomes of bites from the same dog based on different treatment of two cases. Rabies is preventable using effective measures including immediate wound washing, vaccine therapy, and wound infiltration with RIG.

## Additional file

Additional file 1: Multilingual abstract in the five official working languages of the United Nations. (PDF 673 kb)

## Abbreviations

CDC: Center for Disease Control and Prevention
CSF: Cerebrospinal fluid
PCR: Polymerase chain reaction
PEP: Postexposure prophylaxis
RIG: Rabies immunoglobulin
RNA: Ribonucleic acid
RV: Rabies virus
RVNA: Rabies virus neutralizing antibody
WHO: World Health Organization

## References

[1] Quiambao BP, Dytioco HZ, Dizon RM, Crisostomo ME, Laot TM, Teuwen DE (2008). Rabies post-exposure prophylaxis in the Philippines: health status of patients having received purified equine F(ab')(2) fragment rabies immunoglobulin (Favirab). PLoS Negl Trop Dis.

[2] Yin W, Dong J, Tu C, Edwards J, Guo F, Zhou H (2013). Challenges and needs for China to eliminate rabies. Infect Dis Poverty.

[3] Zhu WY, Liang GD (2012). Current status of canine rabies in China. Biomed Environ Sci.

[4] Rudd RJ, Appler KA, Wong SJ (2013). Presence of cross-reactions with other viral encephalitides in the indirect fluorescent-antibody test for diagnosis of rabies. J Clin Microbiol.

[5] Dacheux L, Wacharapluesadee S, Hemachudha T, Meslin FX, Buchy P, Reynes JM (2010). More accurate insight into the incidence of human rabies in developing countries through validated laboratory techniques. PLoS Negl Trop Dis.

[6] Tamura K, Peterson D, Peterson N, Stecher G, Nei M, Kumar S (2011). MEGA5: molecular evolutionary genetics analysis using maximum likelihood, evolutionary distance, and maximum parsimony methods. Mol Biol Evol.

[7] Madhusudana SN, Malavalli BV, Thankappan UP, Sundramoorthy S, Belludi AY, Pulagumbaly SB (2014). Development and evaluation of a new immunohistochemistry-based test for the detection of rabies virus neutralizing antibodies. Hum Vaccin Immunother.

[8] Fang Y, Chen L, Liu MQ, Zhu ZG, Zhu ZR, Hu Q (2014). Comparison of safety and immunogenicity of PVRV and PCECV immunized in patients with WHO category II animal exposure: a study based on different age groups. PLoS Negl Trop Dis.

[9] Si H, Guo ZM, Hao YT, Liu YG, Zhang DM, Rao SQ (2008). Rabies trend in China (1990-2007) and post-exposure prophylaxis in the Guangdong province. BMC Infect Dis.

[10] Gowda VK, Basavaraja GV, Reddy H, Ramaswamy P (2014). Paralytic rabies following cat scratch and intra-dermal anti-rabies vaccination. J Pediatr Neurosci.

[11] Shantavasinkul P, Wilde H (2011). Postexposure prophylaxis for rabies in resource-limited/poor countries. Adv Virus Res.

[12] Abubakar SA, Bakari AG (2012). Incidence of dog bite injuries and clinical rabies in a tertiary health care institution: a 10-year retrospective study. Ann Afr Med.

[13] Stahl JP, Gautret P, Ribadeau-Dumas F, Strady C, Le Moal G, Souala F (2014). Update on human rabies in a dog- and fox-rabies-free country. Med Mal Infect.

[14] Susilawathi NM, Darwinata AE, Dwija IB, Budayanti NS, Wirasandhi GA, Subrata K (2012). Epidemiological and clinical features of human rabies cases in Bali 2008-2010. BMC Infect Dis.

[15] Yu J, Li H, Tang Q, Rayner S, Han N, Guo Z (2012). The spatial and temporal dynamics of rabies in China. PLoS Negl Trop Dis.

[16] Udow SJ, Marrie RA, Jackson AC (2013). Clinical features of dog- and bat-acquired rabies in humans. Clin Infect Dis.

