# Supplementary material for: Bites from the same dog, different outcomes for two patients: a case report
Source: Infect Dis Poverty. 2017 Jul 5;6:107. doi: 10.1186/s40249-017-0321-3 (PMC5497382; doi:10.1186/s40249-017-0321-3)
Supplement: Additional file 1: — Multilingual abstract in the five official working languages of the United Nations. (PDF 673 kb) [file 40249_2017_321_MOESM1_ESM.pdf]

Translation of the abstract into the five official working languages of the United Nations

عضات من نفس الكلب تؤدي لنتائج مختلفة في إثنين من المرضي: تقرير حالة

شوى-يونغ هوانغ، شينغ-لو لي، شو-يو وو، يو-لي غو، شين-جون لوفي، جون دافيد كلينه، بيان-لي شو

الملخص
الخلفية: داء السعار من الأمراض حيوانية المنشأ الخطيرة التي عاودت الظهور مجددا في الصين وفي الوقت الحاضر يتم تشخيص حالات الإصابة البشرية بالسعار مبدئيا استنادا إلى الفحص السريري.
عرض الحالة: في أغسطس 2012، تعرضت امرأه وابنها للهجوم من قبل كلب ضال في هينان بالصين وقد تلقي الابن علاجا وقائيا بعد التعرض لداء السعار (معالجه الجروح متبوعا بالتحصين، عدم وجود غلوبولين مناعي) , غير ان الام لم تتلقي العلاج وكنتيجة تأكدت إصابتها بالسعار وماتت نتيجة ذلك في ديسمبر من نفس العام وظل الابن علي قيد الحياة ابنها على قيد الحياة وبصحة جيدة بعد سنتان من المتابعة.
الاستنتاج: يوثق هذا التقرير ان استخدام المعالجة الوقائية في الوقت المناسب بعد التعرض للعض هو تدبير مهم للوقاية من داء السعار بعد التعرض لعضه حيوانيه.

Translated from English version into Arabic by Mohamed R. Habib

同一条狗咬伤两人而结局不同：一份病例报告

黄学勇，李幸乐，吴蜀豫，谷玉雷，吕新军，John David Klena，许汴利

摘要
引言：在中国，狂犬病是一种再度出现的严重人畜共患病，目前人类狂犬病诊断主要基于病人的临床表现。
病例介绍：2012 年 8 月，在中国河南省一只流浪犬同时咬伤一名妇女和她的儿子。儿子接受了狂犬病暴露后预防措施（伤口处理和狂犬疫苗接种，未注射免疫球蛋白），而他的母亲没有进行任何预防性处理。后来，实验室确诊母亲感染狂犬病病毒并发病，于 12 月死亡。我们对她的儿子进行 2 年的随访，身体状况良好。
结论：该病例报告显示，被可疑狂犬病毒感染动物咬伤后，及时进行暴露后预防处置能够有效预防狂犬病。

Translated from English version into Chinese by Xue-Yong Huang

Morsures d'un même chien, différents effets de deux patients: rapport d’un cas

Xue-Yong Huang, Xing-Le Li, Shu-Yu Wu, Yu-Lei Gu, Xin-Jun Lv, John David Klena, Bian-Li Xu

Résumé
Contexte: La rage est une grave zoonose réémergeante en Chine. À l'heure actuelle, les cas de rage humaine sont principalement diagnostiqués à partir de cas cliniques.
Présentation de cas: En août 2012, une femme et son fils ont été attaqués par un chien errant dans la Province de Henan en Chine. Le fils a reçu un traitement prophylactique post-exposition contre la rage (traitement de blessure suivi du vaccin, pas d'immunoglobuline), cependant, la mère ne l'a pas fait. La rage a été confirmée en laboratoire chez la mère et elle est morte en Décembre; son fils est vivant et en bonne santé après deux ans de suivi.
Conclusion: Le présent article instruit que la prophylaxie post-exposition à temps est une mesure nécessaire pour

prévenir la rage après exposition à une morsure d'animal.

Translated from English version into French by Kokouvi Kassegne

Укусы от той же собаки, разные результаты для двух пациентов: Отчёт случая

Xue-Yong Huang, Xing-Le Li, Shu-Yu Wu, Yu-Lei Gu, Xin-Jun Lv, John David Klena, Bian-Li Xu

Аннотация

Введение: Бешенство - это серьезные зоонозы, которые воссоздавались в Китае. В настоящее время случаи бешенства у людей, в основном, диагностированные на основании клинической картины.

Презентация случая: В августе 2012 года, одна женщина и её сын подверглись нападению бродячих собак в провинции Хэнань, Китай. Сын получил постконтактную профилактику бешенства (после обработки раны сын принял вакцины, без иммуноглобулин), однако, мать не приняла вакцины. Инфекция бешенства позже был лабораторно подтверждена у матери и она умерла в декабре; её сын жив и здоров после двухлетного наблюдения.

Выводы: Данный отчёт свидетельствует о том, что своевременное использование постконтактной профилактики является необходимой мерой по предотвращению бешенства после контакта с укусом животного.

Translated from English version into Russian by Hao-Qi Zhang

Mordeduras del mismo perro, resultados diferentes para dos pacientes: Reporte de un caso

Xue-Yong Huang, Xing-Le Li, Shu-Yu Wu, Yu-Lei Gu, Xin-Jun Lv, John David Klena, Bian-Li Xu

Abstracto

Trasfondo: La rabia es una seria zoonosis reemergente en China. En la actualidad, los casos de rabia humana son diagnosticados principalmente basándose en la presentación clínica.

Presentación del caso: En agosto de 2012, una mujer y su hijo fueron atacados por un perro callejero en Henan, China. El hijo recibió profilaxis post-exposición de la rabia (tratamiento de la herida seguido por la vacuna, no inmunoglobulina), sin embargo, la madre no lo hizo. La infección de la rabia fue posteriormente confirmada por el laboratorio en la madre y murió en diciembre; Su hijo está vivo y sano después de 2 años de seguimiento.

Conclusión: Este informe documenta que la utilización oportuna de la profilaxis post-exposición es una medida necesaria para prevenir la rabia después de la exposición a una mordedura animal.

Translated from English version into Spanish by Laura C Vicente Rodriguez
